# Anaesthesiologists’ guideline adherence in pre-operative evaluation: a retrospective observational study

**DOI:** 10.1186/s13741-024-00424-5

**Published:** 2024-06-28

**Authors:** Simone Maria Kagerbauer, Jennifer Wißler, Manfred Blobner, Ferdinand Biegert, Dimislav Ivanov Andonov, Gerhard Schneider, Armin Horst Podtschaske, Bernhard Ulm, Bettina Jungwirth

**Affiliations:** 1 Department of Anaesthesiology and Intensive Care Medicine, School of Medicine, Technical University of Munich, Munich, Germany; 2 Department of Anaesthesiology and Intensive Care Medicine, School of Medicine, University of Ulm, Albert-Einstein-Allee 23, Ulm, 89081 Germany

**Keywords:** Pre-operative evaluation, Guidelines, Adherence, Decision-making, Decision support

## Abstract

**Background:**

Surveys suggest a low level of implementation of clinical guidelines, although they are intended to improve the quality of treatment and patient safety. Which guideline recommendations are not followed and why has yet to be analysed. In this study, we investigate the proportion of European and national guidelines followed in the area of pre-operative anaesthetic evaluation prior to non-cardiac surgery.

**Methods:**

We conducted this monocentric retrospective observational study at a German university hospital with the help of software that logically links guidelines in such a way that individualised recommendations can be derived from a patient's data. We included routine logs of 2003 patients who visited our pre-anaesthesia outpatient clinic between June 2018 and June 2020 and compared the actual conducted pre-operative examinations with the recommendations issued by the software. We descriptively analysed the data for examinations not performed that would have been recommended by the guidelines and examinations that were performed even though they were not covered by a guideline recommendation. The guidelines examined in this study are the 2018 ESAIC guidelines for pre-operative evaluation of adults undergoing elective non-cardiac surgery, the 2014 ESC/ESA guidelines on non-cardiac surgery and the German recommendations on pre-operative evaluation on non-cardiothoracic surgery from the year 2017.

**Results:**

Performed ECG (78.1%) and cardiac stress imaging tests (86.1%) indicated the highest guideline adherence. Greater adherence rates were associated with a higher ASA score (ASA I: 23.7%, ASA II: 41.1%, ASA III: 51.8%, ASA IV: 65.8%, *P* < 0.001), lower BMI and age > 65 years. Adherence rates in high-risk surgery (60.5%) were greater than in intermediate (46.5%) or low-risk (44.6%) surgery (*P* < 0.001). 67.2% of technical and laboratory tests performed preoperatively were not covered by a guideline recommendation.

**Conclusions:**

Guideline adherence in pre-operative evaluation leaves room for improvement. Many performed pre-operative examinations, especially laboratory tests, are not recommended by the guidelines and may cause unnecessary costs. The reasons for guidelines not being followed may be the complexity of guidelines and organisational issues. A software-based decision support tool may be helpful.

**Trial registration:**

ClinicalTrials.gov ID NCT04843202.

**Supplementary Information:**

The online version contains supplementary material available at 10.1186/s13741-024-00424-5.

## Background

Perioperative morbidity and mortality are associated with insufficient perioperative management (Pearse et al. 2012). Therefore, morbidity and mortality, at least of elective surgery, may be reduced by assessing the patients’ pre-operative health status for risk stratification and identification of best perioperative care (De Hert et al. 2018; Kristensen et al. 2014). The European Society of Anaesthesiology and Intensive Care Medicine (ESAIC) provides uniform standards for pre-operative evaluation. The society has issued clinical guidelines which, although not legally binding, provide a consensually accepted basis to assist anaesthesiologists in their decision-making (De Hert et al. 2018, 2011). Beyond that, in Germany, there are recommendations for action by the German Society of Anaesthesiology and Intensive Care Medicine (DGAI) in addition to the ESAIC guidelines already mentioned, which differ somewhat from the European guidelines (Geldner et al. 2017). These national recommendations are not guidelines in the strict sense of the word but rather a summary of guidelines and good clinical practice. To simplify things, they are further also referred to as guidelines.

In a German survey, a large proportion of anaesthesia department chairs stated that they were aware of the existence of guidelines for pre-operative evaluation, but even those of university hospitals reported a 54% implementation rate only (Bohmer et al. 2012). Even more, it is still unclear what the levels of adherence to implemented guidelines are in clinical practice (Aust et al. 2013). To the best of our knowledge, a status quo analysis of guideline adherence in European countries does not yet exist. To facilitate this analysis in our study, we used software that can be integrated into a patient data management system (PDMS) for structured pre-anaesthesia data collection. This decision support system outputs the guideline recommendations applicable to each individual patient and enables a systematic analysis of which guideline recommendations are followed during the pre-anaesthesia visit. Our hypothesis is that adherence to guidelines is generally low but may depend on certain factors, such as the source of the guideline, the level of recommendation, the patient’s general condition and surgical risk. We also suspect many non-indicated examinations are carried out, especially in patients without pre-existing diseases.

The aim of this exploratory study is, therefore, to gain a picture of compliance with the ESAIC guidelines as well as the German guidelines for pre-operative evaluation and to identify factors for their possible lack of adherence in the clinical practice of a German university hospital.

## Methods

After approval by the institutional ethics committee (Ethics Committee of the Medical Faculty of the Technical University of Munich (TUM), Chairperson Prof G. Schmidt, ethics committee number 450/20 S-EB of 29 June 2020), and Clinical Trials registration (ClinicalTrials.gov ID NCT04843202), this monocentric study was conducted at a German university hospital. Due to the retrospective design of the study, informed consent was waived.

We used the size of a comparable observational study as a measure (Flamm et al. 2011) and chose 2000 patients as a representative sample for a descriptive analysis. This corresponded to about 5% of the total available pre-anaesthesia evaluation protocols from the study period.

Patients were selected using a random algorithm which is provided in the appendix (Supplement C1). Stratification took place according to the frequency of patients in each ASA class of the overall collective. Due to incomplete data entries in about 0.5% of the electronic health records (EHR), we had to stratify 2010 patients in total.

2003 complete records were available for the study. We included adult non-cardiac surgery patients who presented to our anaesthesiologic outpatient clinic between June 2018 and June 2020 for pre-operative assessment prior to elective surgery with a planned postoperative stay in the hospital.

Pre-anaesthesia visit logs were routinely generated in the pre-anaesthesia module of the anaesthesia patient data management system (PDMS) and stored in the EHR. These logs included information collected by the anaesthesiologist during the patient’s pre-operative presentation on medical history, physical complaints, physical examination findings, laboratory values, findings of technical examinations, and the current medication.

These data were analysed with the help of software that has been developed in cooperation with the Department of Anaesthesiology and Intensive Care Medicine of the TUM with HIM (Health Information Management GmbH, Bad Homburg, Germany) as part of a research project. In detail, before software development, specialists in anaesthesiology screened the guidelines and selected the relevant recommendations. Controversies were resolved by a consensus of three experts. The recommendations were then transferred into if/else conditions or logical rules. The input mask for the medical history survey was adapted so that all information required for the guidelines became mandatory questions. This relevant information was requested exclusively with the help of checkboxes, radio buttons or drop-down lists; free text was avoided to have precise, unambiguous answers. Code review was performed by a computer programmer and a specialist in anaesthesiology with basic programming skills. In addition to the code review, the software was tested before the study by two anaesthesiologists who were not involved in the development using case vignettes to check the results of the software for plausibility.

Since the software prototype had no interfaces to the hospital information system, data entry was manually done by an anaesthesiologist assisted by a medical student. The data was entered by two people to enable a cross-check and minimise the risk of incorrect entries. The basis for the entries was the above-described pre-anaesthesia visit logs of the respective patients, which were stored in PDF format in the hospital information system. These documents summarise all pre-operative clinical findings and examinations combining structured data and free text which were transferred manually to the software system. The software displays a synopsis of applicable guideline recommendations at the end which served as a benchmark for guideline adherence in this study.

The origin of the guideline recommendations (ESAIC as the European or DGAI as the national society) is also displayed alongside the recommendation text issued by the software. Guidelines included are the 2018 ESAIC guidelines for pre-operative evaluation of adults undergoing elective non-cardiac surgery (De Hert et al. 2018) supplemented by the 2014 ESC/ESA guidelines on non-cardiac surgery (Kristensen et al. 2014) and the German recommendations on pre-operative evaluation on non-cardiothoracic surgery (Geldner et al. 2017).

ESAIC guideline recommendations are classified according to the GRADE system and are graded by level of recommendation (strong or weak) and level of evidence (high, moderate, or low quality of evidence) (De Hert et al. 2018). This classification is additionally displayed if an ESAIC guideline recommendation applies. There is no such categorisation for the recommendations of the German specialist society. Concrete recommendations for action described in the text of the ESAIC guidelines without being assigned a level of evidence are also included in the analysis. There is an overlap between the DGAI and ESAIC recommendations, which are partially identical. In such cases, the software issues the recommendation only once, but assigns both labels ‘ESAIC’ and ‘DGAI’.

In the context of this study, we restricted the assessment of guideline adherence exclusively to technical examinations (laboratory values, electrocardiogram (ECG), echocardiography, pulmonary function testing, carotid Doppler, non-invasive stress testing, and coronary angiography). We did not include cardiac biomarkers in our analysis, because biomarker testing was not yet established at the time of the study in our hospital.

Pre-anaesthesia assessments in Germany are always performed by physicians who are either residents or specialists in anaesthesiology. No electronic decision support tool was available for the standard evaluation at our pre-anaesthesia outpatient clinic during the study period.

To analyse guideline adherence, we compared the actual examinations performed according to the original protocol of the pre-anaesthesia visit patient for patient with the recommendations issued by the software. Depending on whether or not a recommendation that was applicable according to the guidelines was followed during the actual pre-anaesthesia visit, we divided the additional examinations performed or recommended into three categories: ‘recommended and DONE’ (a guideline recommendation applied to the patient and was followed), ‘recommended and NOT done’ (a guideline recommendation that applied was not followed) and ‘not recommended BUT done’ (an examination was ordered by the physician and performed without being covered by a guideline recommendation). We defined guideline adherence as the ratio of the number of followed recommendations to the number of all recommendations issued:

$$\frac{\mathrm{number}\;\mathrm{of}\;'\mathrm{recommended}\;\mathrm{and}\;\mathrm{DONE}'}{\mathrm{number}\;\mathrm{of}\;'\mathrm{recommended}\;\mathrm{and}\;\mathrm{DONE}'\;+\;\mathrm{number}\;\mathrm{of}\;'\mathrm{recommended}\;\mathrm{and}\;\mathrm{NOT}\;\mathrm{done}'}$$

Depending on the clinical conditions, an additional examination could be recommended in a patient several times by different guidelines. For example, an ECG may be required because of age or—regardless of age—because of pre-existing conditions. Pre-operative examinations that were ‘not recommended BUT done’ can commonly not be assigned to a recommendation. They were therefore analysed separately and assessed as not indicated.

To provide a clear overview, all guideline recommendations were divided into 17 subgroups: recommendations requiring ECG, echocardiography, pulmonary function testing, non-invasive cardiac stress testing, carotid doppler, coronary angiography, pulse oximetry, and several laboratory examinations which we regarded separately. A differentiated analysis of the guideline recommendations according to ESAIC and national guidelines was conducted, as well as one merging both sources. The ESAIC guidelines were additionally evaluated according to the grade of recommendation and level of evidence. Guideline adherence according to certain patient characteristics was analysed according to ASA, body-mass index, age and surgical risk. For analysis, patients were divided into body mass index groups according to the definition of the World Health Organization: below 18.5 was assigned underweight, 18.5-below 25 normal weight, 25-below 30 overweight and 30 or more obese (https://www.who.int ) (WHO n.d.).

In addition, the proportion of not indicated examinations (‘not recommended BUT done’) was also determined for each technical and laboratory examination.

Descriptive statistics was performed using R version 4.2.2 (R Foundation for Statistical Computing, Vienna, Austria). *χ*2 test and post-hoc pairwise Fisher test or test of given proportions were used to detect differences between patient groups and guideline adherence. *P* < 0.05 was considered statistically significant.

## Results

### Patient characteristics

Two thousand three patient records were included in the study, 42.7% of patients were female, the mean age was 55.4 ± 17.8 years, and the mean BMI was 26.3 ± 4.9 kg m^−2^. In the collective studied, 21.6% of patients were ASA class I, 44.8% ASA II, 32.7% ASA III, and 1.0% ASA IV which corresponded to the actual distribution of the ASA classes at our hospital. Details are shown in Table 1.

**Table 1:** Patient characteristics

Characteristic	*n* (sum = 2003)	
Sex		
Female	855	43%
Male	1.148	57%
Age (years)		
< 65	1.287	64%
≥ 65	716	36%
BMI		
Underweight	46	2%
Normal	737	37%
Overweight	798	40%
Obese	422	21%
Surgical risk^a^		
Low (< 1%)	948	47%
Intermediate (1–5%)	1.008	50%
High (> 5%)	47	2%
ASA		
I	432	22%
II	897	45%
III	654	33%
IV	20	1%
Smoker		
No	1.185	59%
Yes	404	20%
Unknown	414	21%
Alcohol abuse		
No	1.338	67%
Yes	155	8%
Unknown	510	25%
Revision surgery		
No	1.852	92%
Yes	151	8%
Major complications after surgery		
No	1.979	99%
Yes	24	1%
In hospital death		
No	2.002	100%
Yes	1	0%

*BMI* body mass index (categories according to WHO classification); major complications include postoperative need for ventilation or catecholamine therapy > 12 h, coagulopathy, sepsis or kidney injury requiring dialysis which result in complex intensive care unit treatment

^a^30-day risk of cardiovascular death and myocardial infarction (surgical risk modified according to Glance et al. (2012))

### Guideline adherence by source and level of recommendation

In total, 9743 individual recommendations were issued by the software, 7923 of which originated from the ESAIC. Overall adherence was 47%.

Considering the guidelines of all professional societies together, ECG (78.1%) and cardiac stress imaging tests (86.1%) had the highest guideline adherence among the technical examinations. Echocardiography showed moderate guideline adherence (53.7%) whereas other technical examinations showed poor guideline adherence (pulse oximetry: 11.3%, carotid Doppler: 7.3%, pulmonary function: 4.3%, cardiac catheterisation: 0%). The best adherence rate for laboratory values was achieved for blood coagulation tests (72.8%) and haemoglobin concentration (71.2%). Particularly poor guideline adherence could be seen in blood glucose tests (22.2%), HbA1c (glycated haemoglobin, 0%) and serum protein (0%).

When looking at the ESAIC and the national guidelines separately, a very similar picture emerges: adherence to laboratory tests and ECG is mostly good but weak for more complex technical examinations.

**Fig. 1:**
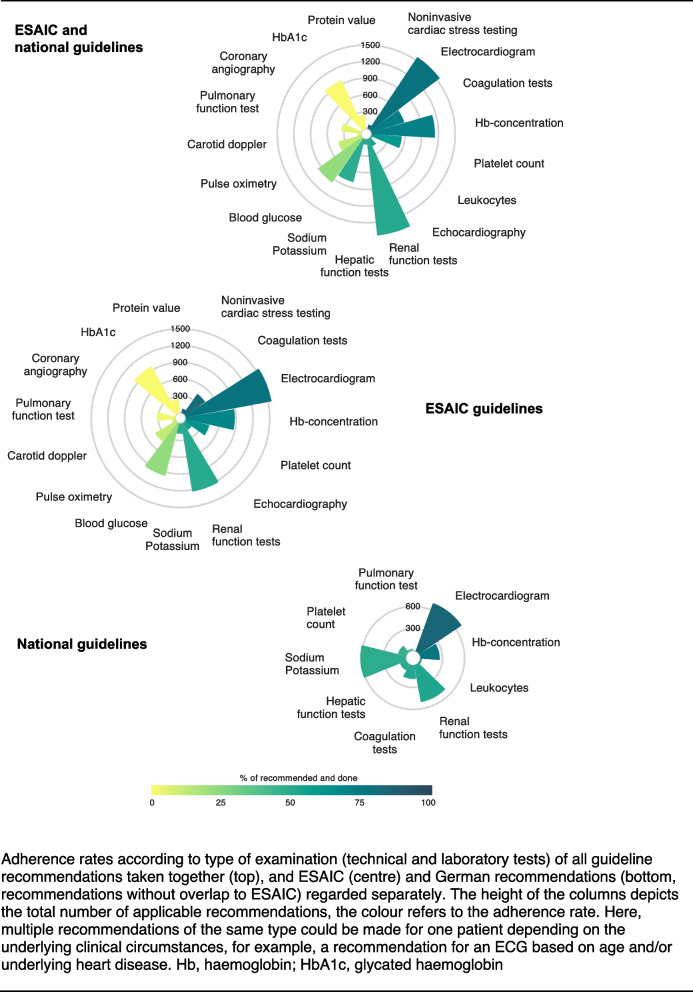
Adherence rates according to type of examination

Figure 1 shows guideline adherence of all recommendations taken together as well as ESAIC and national recommendations separately. A detailed tabular overview is provided in Additional Table A1.

ESAIC guidelines were additionally analysed by recommendation levels. In addition to recommendations that were graded 1A-C or 2A-C, we included recommendations for action that were detailed in the long text of the guidelines but were not explicitly assigned a recommendation grade. Overall, 1B recommendations showed the lowest adherence. Guideline adherence was even lower in class 1 recommendations compared with class 2 recommendations (*P* < 0.01). Details are depicted in Table 2.

**Table 2:** ESA guideline adherence by recommendation grade

	Recommendation grade													
	1A		1B		1C		2A		2B		2C		None	
Recommendations	*n*	Adherence	*n*	Adherence	*n*	Adherence	*n*	Adherence	*n*	Adherence	*n*	Adherence	*n*	Adherence
Total	15	53%	75	16%	329	75%	1516	26%	2639	42%	724	51%	4445	54%
Technical examinations														
ECG			75	16%	163	85%			645	82%			676	80%
Echocardiography	15	53%			73	70%			3	0%	47	28%	11	73%
Pulmonary function test									3	0%			349	4%
Non-invasive cardiac stress testing							2	0%	67	93%			3	0%
Carotid doppler									55	7%				
Coronary angiography									15	0%				
Pulse oximetry									424	11%				
Laboratory values														
Sodium/Potassium													798	49%
Hb-concentration					2	100%			579	68%			553	75%
Leukocytes													20	55%
Platelet count							430	62%					112	47%
Coagulation tests											430	82%	184	51%
Renal function tests					91	63%							1647	50%
Hepatic function tests													92	50%
Protein value											247	0%		
Blood glucose							542	25%	424	19%				
HbA1c							542	0%	424	0%				

*n* total number of recommendations, *Adherence* adherence rate in %, *None* good clinical practice recommendations mentioned in the text without being assigned a recommendation grade, *ECG* electrocardiogram, *HbA1c* glycated haemoglobin, *Hb* haemoglobin

### Guideline adherence according to patient condition and surgical risk

**Fig. 2:**
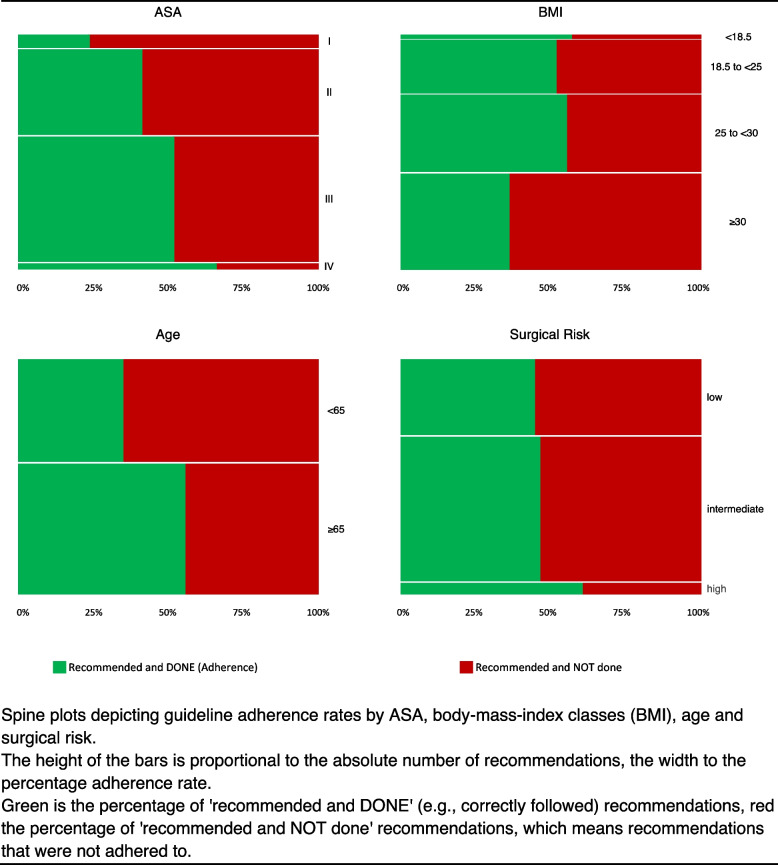
Spine plots of guideline adherence rates

Regarding guideline adherence as reflected by the proportion of ‘recommended and DONE’ recommendations, we additionally analysed subgroups of the study population, the main findings of which are summarised in Fig. 2.

In summary, better adherence rates were associated with a higher ASA score (ASA I 23.7%, ASA II 41.1%, ASA III 51.8%, ASA IV 65.8%, *P* < 0.001). This observation applies especially to the technical examinations of echocardiography, ECG and cardiologic stress tests, and laboratory tests. The increase in guideline adherence with increasing ASA score is especially evident in the blood sugar tests, haemoglobin concentration, renal function tests, electrolytes, coagulation tests and platelet count. Details are shown in Additional Table A2.

A lower BMI showed better adherence rates than a high one (underweight 57.0%, normal 51.8%, overweight 55.3%, obese 36.2%). Statistical significance could be shown for the normal versus obese, obese versus overweight and obese versus underweight groups (*P* < 0.001). Here it is noticeable that pulmonary function tests were mostly performed in overweight but less often in obese patients. Non-invasive cardiac stress testing was performed in the majority of patients when indicated. Guideline adherence by BMI is shown in additional Table A3.

Regarding age, it is evident that better adherence rates are present in the group of 65 years or older (55.6%) than in the group under 65 years (35.0%, *P* < 0.001). When observing the subgroups, this is primarily shown in the technical examinations, especially echocardiography and ECG. For the laboratory values, adherence rates also tend to be greater in the age group over 65 years (additional Table A4).

Adherence rates in high-risk surgery (60.5%) were greater than in intermediate (46.5%) or low risk (44.6%) surgery (*P* < 0.001) (Glance et al. 2012). No significant difference could be found between the low and intermediate-risk surgery groups. Regarding technical examinations, adherence was acceptable for ECG, cardiac stress tests and pulmonary function tests, but a poorer adherence rate was shown for echocardiography. Almost all laboratory tests show a better compliance rate with increasing surgical risk (additional Table A5).

### Not indicated examinations

**Fig. 3:**
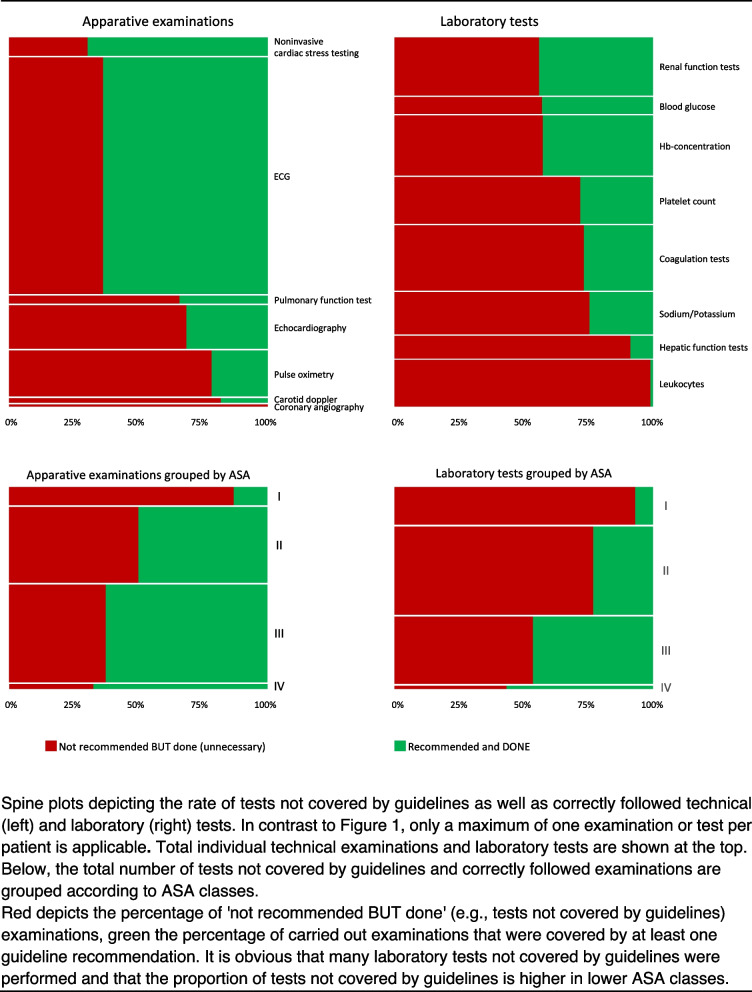
Spine plots depicting the rates of examinations not covered by guidelines

When all of the technical and laboratory tests performed were considered, 67.2% of them were not covered by a guideline recommendation. In most cases, this concerned laboratory tests. Examinations that were performed despite not being recommended by the guidelines occurred mostly in ASA classes I and II. The proportion of these presumably not indicated examinations was significantly higher in ASA class I than in the other three ASA classes (*P* < 0.001) and higher in ASA class II compared with ASA III and IV (*P* < 0.01). Details are shown in Fig. 3 and additional Table A6.

## Discussion

In the present study, we showed that guideline adherence to European and national recommendations concerning pre-anaesthesia evaluation of adults undergoing non-cardiac surgery leaves room for improvement. Adherence rates vary widely and are better in patients with a higher ASA risk class, age > 65 years, in the absence of obesity and with higher surgical risk. A considerably high number of examinations not covered by guidelines are ordered prior to surgery. Our results are consistent with the self-assessment of German anaesthesiologists where almost 40% of respondents admitted to not having sufficient guideline knowledge (Aust et al. 2013). However, lack of adherence to the guidelines may be caused not only by differences in individual knowledge of the anaesthesia staff but also by lack of enforcement, lack of training of the disciplines involved in the perioperative process, and obstacles due to the complexity of the organisational processes (Hoorn et al. 2019).

The impact of physicians’ knowledge, the level of training and adequate resources on guideline adherence can account for some findings of this study. The easier availability of the German guidelines, for instance, may explain why anaesthesiologists in our study are more familiar with their national recommendations than with those of the ESAIC. In addition, the national guidelines are shorter and less comprehensive than the ESAIC guidelines and seem to be more focused on clinical applicability, e.g. actions that can still be performed before surgery to improve patient condition. Furthermore, the native language seems to provide easier access to the information. On the other hand, the higher adherence rates in patients of higher ASA risk classes (Hackett et al. 2015), in elderly patients, and in patients scheduled for procedures with a high risk of perioperative cardiac complications (Glance et al. 2012) suggests that anaesthesiologists attached more importance to a thorough risk evaluation when it seems to matter.

The results of our study indicate that the focus of pre-operative evaluation is very much on cardiac assessment: recommended ECGs and non-invasive stress testing are performed in most cardiac-risk patients. In contrast, metabolic diseases may often not be perceived as a risk: HbA1c values in diabetic patients or serum protein levels in malnourished patients were not determined in a single case.

Therefore, it is obviously a management task to demand care not only for cardiac high-risk patients. A good example is the blanket disregard of the recommendation for pulse oximetry despite the presence of equipment in the anaesthesiology outpatient department. It was therefore only consequent that the documentation of a pulsoxymetrically measured oxygen saturation, similar to blood pressure and pulse rate, was defined as a mandatory field of the pre-operative anaesthesia log of the department. Integrated into an appropriate interprofessional and digitalised environment, such measures can potentially help to mitigate the increased staffing and time required for guideline-compliant procedures that physicians often complain about (Bohmer et al. 2012).

Another reason for the non-compliance with guidelines may also lie in the shared responsibility for the pre-operative process (Baron et al. 2017). Therefore, it is crucial that not only anaesthesiologists but also surgeons know the respective guidelines and implement them in a coordinated manner. This starts most simply with a common understanding of the necessary time between pre-operative evaluation and elective surgery, especially for the medically necessary and sometimes elaborate pre-operative measures, and not just for legal considerations (Geldner et al. 2017). The importance of time pressure is evident in this study regarding adherence rates for examinations that are particularly time-consuming and organisationally demanding and may probably not lead to patient improvement before surgery. For example, recommendations concerning transthoracic echocardiography are followed only moderately, or others, such as carotid Doppler, pulmonary function testing and cardiac catheterisation, are followed poorly.

The traditional approach to pre-operative diagnostics has been to use standardised lists of laboratory tests and technical examinations used by clinicians and general practitioners, who often assume that a standard preoperative laboratory and ECG are mandatory for every patient. We must point out that the software used in this study does not allow us to differentiate between laboratory values determined in the hospital and those brought in by the general practitioner. Findings brought in by the general practitioner have, for sure, contributed to a certain extent to the not indicated laboratory tests. Although the individual examination only accounts for a small amount, non-indicated laboratory tests and non-indicated ECGs cause high costs in total, which have been described in former studies (Flamm et al. 2011). An additional minor factor for over-analysis is the application of automated laboratory tests, which often do not allow analysis of a single parameter (e.g. sodium/potassium) but will provide a whole set of parameters that come with it.

Another aspect that needs to be discussed is that the guideline recommendations only deal with specific preoperative issues. For example, when taking medication that can cause kidney or liver damage or blood count changes, a laboratory test may be medically justified without finding a counterpart in the guidelines for preoperative evaluation. Due to the retrospective study design, this is always a case-by-case decision for the individual patient and cannot be verified for every case.

On the other hand, it must also be mentioned that many preoperative laboratory tests recommended by the guidelines were not performed. This mainly concerns tests that are not included in the standard profiles, such as HbA1c or protein levels. It is particularly striking that not a single diabetic in our patient group had an HbA1c value documented in the records or requested by the anaesthesiologist. However, it must be said that at the time of the study, it was only a weak recommendation, which has been upgraded to a strong recommendation in the current ESC guidelines.

However, poor adherence to guidelines often results from a combination of the above reasons, which can be vividly illustrated by the disregard for obesity-related recommendations. It probably started with the fact that not every BMI > 30 kg m^−2^ was recognised as long as the entry of height and weight into the anaesthesia log was not mandatory and the BMI was not displayed. Furthermore, blood glucose, HbA1c and pulmonary function tests, which are frequently requested by guideline recommendations for these patients, are not part of routine lists. Finally, it is often unlikely that clinical consequences will be drawn from these findings because most of the established measures would rarely be effective in the time available until surgery, especially as these were often weak recommendations according to the GRADE system (Guyatt et al. 2011).

### Strengths and limitations

Our study has limitations due to the retrospective design. Data were taken from the visit logs before anaesthesia, but no study-related patient interview was conducted. As a result, under-documentation, especially of examinations already performed in the outpatient setting, but also of non-pathological findings, is likely. Such missing information may also have influenced the retrospective assessment of guideline adherence. Furthermore, and despite the agreement with similar rates of guideline adherence of 50–60% (Bohmer et al. 2012), we report from only one centre from a German university hospital.

It is a strength of the study that in our pre-anaesthesia outpatient clinic, an electronic documentation system guides doctors through the pre-anaesthetic assessment in a structured way. However, electronic decision support is not used during the standard pre-anaesthesia visit. To give sufficient time to disseminate the guidelines published between October 2014, June 2017, and February 2018 we started the survey in June 2018.

Regarding the software tool used, the implementation of guideline recommendations was performed using simple control structures such as conditional statements and branches. If the corresponding conditions are met and correctly entered into the system, we assume a correct output of the applicable guideline recommendations. This was tested using case vignettes before conducting the study. However, a further requirement for the software is the creation of a data set providing the required information in structured form. The rigorous avoidance of free text in the data input form is a possible source of bias and might have led to information loss, especially as data entry was performed manually, and thus to an inconsistent implementation of the guidelines on the part of the software.

It has to be taken into account that guideline adherence is difficult to determine, and there is no unique definition of it. Often, only the adherence rate to grade 1A recommendations is taken as a benchmark (Kentenich et al. 2023). The strength of our study is that we used a software-guided approach which enabled us to analyse every guideline recommendation. As a drawback of this approach, weaker recommendations may have biased the results towards lower adherence.

## Conclusions

Our study shows that the implementation of guidelines in pre-operative evaluation leaves room for improvement. Recommended examinations that are not performed, as well as non-recommended examinations that take place, may limit the quality of care and lead to unnecessary costs. The most important thing for successful guideline implementation is that the physician knows the guidelines and considers them useful (Bohmer et al. 2014). In addition, the organisational conditions for consistent implementation of the guidelines must be optimised and reasons for the non-adherence need to be further investigated.

## Supplementary Information

Supplementary Material 1: Table A1. Guideline recommendations and adherence rates of all recommendations and of ESAIC and national guideline recommendations. Table A2. Guideline adherence by ASA score. Table A3. Guideline adherence by BMI. Table A4. Guideline adherence by age. Table A5. Guideline adherence by surgical risk. Table A6. Examinations not covered by guidelines. Supplement C1. Patient randomisation algorithm (R Version 4.2.2).

## Data Availability

Due to legal requirements, we are not allowed to store data, although it is de-identified, in a publicly accessible repository. To gain access, proposals should be directed to the corresponding author. Requestors will need to sign a data access agreement.

## Abbreviations

ASA: American Society of Anesthesiologists’ physical score
DGAI: German Society of Anaesthesiology and Intensive Care Medicine
EHR: Electronic health record
ESAIC: European Society of Anaesthesiology and Intensive Care Medicine
GRADE: Grades of Recommendation, Assessment, Development, and Evaluation
PDMS: Patient data management system
