# Supplementary material for: Anaesthesiologists’ guideline adherence in pre-operative evaluation: a retrospective observational study
Source: Perioper Med (Lond). 2024 Jun 28;13:64. doi: 10.1186/s13741-024-00424-5 (PMC11542447; doi:10.1186/s13741-024-00424-5)
Supplement: Supplementary file 1 — Supplementary Material 1: Table A1. Guideline recommendations and adherence rates of all recommendations and of ESAIC and national guideline recommendations. Table A2. Guideline adherence by ASA score. Table A3. Guideline adherence by BMI. Table A4. Guideline adherence by age. Table A5. Guideline adherence by surgical risk. Table A6. Examinations not covered by guidelines. Supplement C1. Patient randomisation algorithm (R Version 4.2.2). [file 13741_2024_424_MOESM1_ESM.docx]

# Supplemental Material

| Table A1: Guideline recommendations and adherence rates of all recommendations and of ESAIC and national guideline recommendations | | | | | | |
| --- | --- | --- | --- | --- | --- | --- |
| Recommended interventions and diagnostics | All recommendations (ESA and German national guidelines) | | Recommendations ESA guidelines | | Recommendations German national guidelines | |
|  | *n* | Adherence | *n* | Adherence | *n* | Adherence |
| Total | 9743 | 47% | 7923 | 45% | 2513 | 62% |
| Technical examinations |  |  |  |  |  |  |
| ECG | 1559 | 78% | 1559 | 78% | 693 | 84% |
| Echocardiography | 149 | 54% | 149 | 54% |  |  |
| Pulmonary function test | 352 | 4% | 326 | 2% | 26 | 35% |
| Noninvasive cardiac stress testing | 72 | 86% | 72 | 86% |  |  |
| Carotid doppler | 55 | 7% | 55 | 7% |  |  |
| Coronary angiography | 15 | 0% | 15 | 0% |  |  |
| Pulse oximetry | 424 | 11% | 424 | 11% |  |  |
| Laboratory values |  |  |  |  |  |  |
| Sodium/Potassium | 798 | 49% | 181 | 50% | 617 | 48% |
| Hb-concentration | 1134 | 71% | 873 | 69% | 261 | 77% |
| Leukocytes | 20 | 55% |  |  | 20 | 55% |
| Platelet count | 542 | 59% | 430 | 62% | 112 | 47% |
| Coagulation tests | 614 | 73% | 430 | 82% | 184 | 51% |
| Renal function tests | 1738 | 51% | 1230 | 50% | 508 | 52% |
| Hepatic function tests | 92 | 50% |  |  | 92 | 50% |
| Protein value | 247 | 0% | 247 | 0% |  |  |
| Blood glucose | 966 | 22% | 966 | 22% |  |  |
| HbA1c | 966 | 0% | 966 | 0% |  |  |
| *n* absolute number of recommendations applicable, *Adherence* adherence rate in %, *ECG* electrocardiogram, *Hb* haemoglobin, *HbA1c* glycated haemoglobin | | | | | | |

| Table A2: Guideline adherence by ASA score | | | | | | | | |
| --- | --- | --- | --- | --- | --- | --- | --- | --- |
| Recommendations | ASA I | | ASA II | | ASA III | | ASA IV | |
|  | *n* | Adherence | *n* | Adherence | *n* | Adherence | *n* | Adherence |
| Total | 565 | 24% | 3619 | 41% | 5299 | 52% | 260 | 66% |
| Technical examinations |  |  |  |  |  |  |  |  |
| ECG | 26 | 62% | 556 | 76% | 936 | 80% | 41 | 73% |
| Echocardiography | 1 | 0% | 29 | 31% | 109 | 58% | 10 | 80% |
| Pulmonary function test | 43 | 0% | 159 | 1% | 142 | 8% | 8 | 13% |
| Noninvasive cardiac stress testing |  |  | 7 | 100% | 57 | 84% | 8 | 88% |
| Carotid doppler | 2 | 0% | 12 | 8% | 39 | 8% | 2 | 0% |
| Coronary angiography |  |  | 5 | 0% | 10 | 0% |  |  |
| Pulse oximetry | 58 | 9% | 205 | 9% | 155 | 14% | 6 | 50% |
| Laboratory values |  |  |  |  |  |  |  |  |
| Sodium/Potassium | 20 | 40% | 195 | 48% | 553 | 48% | 30 | 70% |
| Hb-concentration | 86 | 69% | 437 | 67% | 577 | 73% | 34 | 91% |
| Leukocytes |  |  | 8 | 50% | 12 | 58% |  |  |
| Platelet count | 19 | 63% | 162 | 65% | 346 | 55% | 15 | 80% |
| Coagulation tests | 23 | 78% | 184 | 71% | 391 | 73% | 16 | 88% |
| Renal function tests | 16 | 38% | 613 | 54% | 1065 | 48% | 44 | 73% |
| Hepatic function tests | 4 | 50% | 30 | 30% | 57 | 60% | 1 | 100% |
| Protein value | 15 | 0% | 115 | 0% | 108 | 0% | 9 | 0% |
| Blood glucose | 126 | 6% | 451 | 14% | 371 | 36% | 18 | 61% |
| HbA1c | 126 | 0% | 451 | 0% | 371 | 0% | 18 | 0% |
| *n* absolute number of recommendations applicable, *Adherence* adherence rate in %, *ECG* electrocardiogram, *Hb* haemoglobin, *HbA1c* glycated haemoglobin | | | | | | | | |

| Table A3: Guideline adherence by BMI | | | | | | | | | |
| --- | --- | --- | --- | --- | --- | --- | --- | --- | --- |
| Recommendations | underweight | | normal | | overweight | | obese | | |
|  | *n* | Adherence | *n* | Adherence | *n* | Adherence | *n* | Adherence | |
| Total | 179 | 57% | 2260 | 52% | 3253 | 55% | 4051 | 36% | |
| Technical examinations |  |  |  |  |  |  |  |  | |
| ECG | 28 | 79% | 420 | 78% | 675 | 80% | 436 | 74% | |
| Echocardiography | 3 | 33% | 52 | 50% | 58 | 50% | 36 | 67% | |
| Pulmonary function test | 1 | 0% | 10 | 20% | 14 | 36% | 327 | 2% | |
| Noninvasive cardiac stress testing | 3 | 100% | 16 | 100% | 27 | 74% | 26 | 88% | |
| Carotid doppler | 3 | 0% | 16 | 6% | 27 | 11% | 9 | 0% | |
| Coronary angiography |  |  | 6 | 0% | 6 | 0% | 3 | 0% | |
| Pulse oximetry |  |  |  |  |  |  | 424 | 11% | |
| Laboratory values |  |  |  |  |  |  |  |  | |
| Sodium/Potassium | 16 | 63% | 228 | 44% | 333 | 53% | 221 | 46% | |
| Hb-concentration | 19 | 84% | 220 | 72% | 295 | 73% | 600 | 70% | |
| Leukocytes | 1 | 0% | 8 | 50% | 8 | 75% | 3 | 33% | |
| Platelet count | 17 | 65% | 151 | 60% | 239 | 61% | 135 | 53% | |
| Coagulation tests | 18 | 78% | 171 | 74% | 267 | 75% | 158 | 68% | |
| Renal function tests | 40 | 58% | 566 | 48% | 716 | 53% | 416 | 49% | |
| Hepatic function tests | 2 | 0% | 28 | 64% | 36 | 42% | 26 | 50% | |
| Protein value | 6 | 0% | 76 | 0% | 92 | 0% | 73 | 0% | |
| Blood glucose | 11 | 18% | 146 | 19% | 230 | 28% | 579 | 21% | |
| HbA1c | 11 | 0% | 146 | 0% | 230 | 0% | 579 | 0% | |
| *n* absolute number of recommendations applicable, *Adherence* adherence rate in %, *ECG* electrocardiogram, *Hb* haemoglobin, *HbA1c* glycated haemoglobin | | | | | | | | |  |

| Table A4: Guideline adherence by age | | | | |
| --- | --- | --- | --- | --- |
| Recommendations | age <65 | | age ≥65 | |
|  | *n* | Adherence | *n* | Adherence |
| Total | 4287 | 35% | 5456 | 55,6% |
| Technical examinations |  |  |  |  |
| ECG | 402 | 65% | 1157 | 82,5% |
| Echocardiography | 37 | 38% | 112 | 58,9% |
| Pulmonary function test | 224 | 1% | 128 | 9,4% |
| Noninvasive cardiac stress testing | 18 | 94% | 54 | 83,3% |
| Carotid doppler | 18 | 0% | 37 | 10,8% |
| Coronary angiography | 4 | 0% | 11 | 0,0% |
| Pulse oximetry | 268 | 10% | 156 | 13,5% |
| Laboratory values |  |  |  |  |
| Sodium/Potassium | 281 | 39% | 517 | 54,0% |
| Hb-concentration | 567 | 66% | 567 | 76,2% |
| Leukocytes | 5 | 20% | 15 | 66,7% |
| Platelet count | 178 | 54% | 364 | 61,3% |
| Coagulation tests | 226 | 65% | 388 | 77,6% |
| Renal function tests | 763 | 44% | 975 | 55,5% |
| Hepatic function tests | 53 | 45% | 39 | 56,4% |
| Protein value | 147 | 0% | 100 | 0,0% |
| Blood glucose | 548 | 16% | 418 | 29,9% |
| HbA1c | 548 | 0% | 418 | 0,0% |
| *n* absolute number of recommendations applicable, *Adherence* adherence rate in %, *ECG* electrocardiogram, *Hb* haemoglobin, *HbA1c* glycated haemoglobin | | | | |

| Table A5: Guideline adherence by surgical risk | | | | | | |
| --- | --- | --- | --- | --- | --- | --- |
| Recommendations | Surgical risk^1^ | | | | | |
|  | low (<1%) | | intermediate (1-5%) | | high (>5%) | |
|  | *n* | Adherence | *n* | Adherence | *n* | Adherence |
| Total | 3187 | 44,6% | 6079 | 46% | 477 | 61% |
| Technical examinations | | | | | | |
| ECG | 457 | 71,1% | 1032 | 81% | 70 | 86% |
| Echocardiography | 30 | 66,7% | 69 | 64% | 50 | 32% |
| Pulmonary function test | 142 | 1,4% | 179 | 1% | 31 | 35% |
| Noninvasive cardiac stress testing | 2 | 0,0% | 66 | 88% | 4 | 100% |
| Carotid doppler |  |  | 41 | 7% | 14 | 7% |
| Coronary angiography | 8 | 0,0% | 7 | 0% |  |  |
| Pulse oximetry | 184 | 14,1% | 233 | 9% | 7 | 0% |
| Laboratory values | | | | | | |
| Sodium/Potassium | 318 | 45,0% | 436 | 49% | 44 | 70% |
| Hb-concentration | 495 | 67,7% | 602 | 72% | 37 | 97% |
| Leukocytes | 11 | 54,5% | 8 | 50% | 1 | 100% |
| Platelet count | 201 | 56,2% | 313 | 58% | 28 | 93% |
| Coagulation tests | 215 | 77,2% | 363 | 70% | 36 | 72% |
| Renal function tests | 495 | 47,7% | 1168 | 51% | 75 | 69% |
| Hepatic function tests | 25 | 24,0% | 58 | 59% | 9 | 67% |
| Protein value | 110 | 0,0% | 124 | 0% | 13 | 0% |
| Blood glucose | 247 | 17,8% | 690 | 22% | 29 | 66% |
| HbA1c | 247 | 0,0% | 690 | 0% | 29 | 0% |
| *n* absolute number of recommendations applicable, *Adherence* adherence rate in %, *ECG* electrocardiogram, *Hb* haemoglobin, *HbA1c* glycated haemoglobin  ^1^Surgical risk modified according to:  Glance LG, Lustik SJ, Hannan EL, Osler TM, Mukamel DB, Qian F, et al. The Surgical Mortality Probability Model:  derivation and validation of a simple risk prediction rule for non-cardiac surgery. Ann Surg. 2012;255(4):696-702. | | | | | | |

| Table A6: Examinations not covered by guidelines | | | | | | | | | | |
| --- | --- | --- | --- | --- | --- | --- | --- | --- | --- | --- |
| Examinations | All Examinations | | ASA I | | ASA II | | ASA III | | ASA IV | |
|  | Done | Not  indicated | Done | Not  indicated | Done | Not  indicated | Done | Not  indicated | Done | Not  indicated |
| Total | 9938 | 67% | 1727 | 93% | 4357 | 73% | 3682 | 50% | 172 | 41% |
| Technical examinations |  |  |  |  |  |  |  |  |  |  |
| ECG | 1163 | 36% | 105 | 86% | 532 | 43% | 512 | 20% | 14 | 7% |
| Echocardiography | 213 | 69% | 6 | 100% | 44 | 82% | 152 | 64% | 11 | 55% |
| Pulmonary function test | 38 | 66% | 0 |  | 9 | 78% | 28 | 64% | 1 | 0% |
| Noninvasive cardiac stress testing | 89 | 30% | 0 |  | 9 | 22% | 72 | 33% | 8 | 13% |
| Carotid doppler | 22 | 82% | 1 | 100% | 5 | 80% | 15 | 80% | 1 | 100% |
| Coronary angiography | 9 | 100% | 0 |  | 1 | 100% | 6 | 100% | 2 | 100% |
| Pulse oximetry | 226 | 78% | 50 | 88% | 78 | 76% | 92 | 77% | 6 | 50% |
| Laboratory values |  |  |  |  |  |  |  |  |  |  |
| Sodium/Potassium | 966 | 75% | 207 | 97% | 447 | 85% | 297 | 48% | 15 | 27% |
| Hb-concentration | 1368 | 57% | 290 | 80% | 610 | 62% | 449 | 38% | 19 | 16% |
| Leukocytes | 1060 | 99% | 229 | 100% | 487 | 99% | 328 | 98% | 16 | 100% |
| Platelet count | 1065 | 72% | 231 | 95% | 491 | 79% | 327 | 46% | 16 | 31% |
| Coagulation tests | 1493 | 73% | 309 | 94% | 659 | 82% | 506 | 50% | 19 | 32% |
| Renal function tests | 1327 | 56% | 206 | 97% | 644 | 59% | 456 | 34% | 21 | 29% |
| Hepatic function tests | 515 | 91% | 60 | 97% | 217 | 96% | 225 | 85% | 13 | 92% |
| Blood glucose | 384 | 57% | 33 | 82% | 124 | 59% | 217 | 53% | 10 | 40% |
| *Done* total number of performed technical or laboratory examinations, *Not indicated* Proportion of examinations that were not covered by a guideline recommendation | | | | | | | | | | |

**Supplement C1**

**Patient randomisation algorithm (R Version 4.2.2):**

#####

# Patientenauswahl

#####

ASA <- Fallnummer_daten %>% filter(FIELD_NAME=="ASA")

prop.table(table(ASA$FIELD_VALUE))

round(2000* prop.table(table(ASA$FIELD_VALUE))[1],0)

round(2000* prop.table(table(ASA$FIELD_VALUE))[2],0)

round(2000* prop.table(table(ASA$FIELD_VALUE))[3],0)

round(2000*(prop.table(table(ASA$FIELD_VALUE))[4]

+prop.table(table(ASA$FIELD_VALUE))[5]

+prop.table(table(ASA$FIELD_VALUE))[5]),0)

sum(round(2000* prop.table(table(ASA$FIELD_VALUE))[1],0),

round(2000* prop.table(table(ASA$FIELD_VALUE))[2],0),

round(2000* prop.table(table(ASA$FIELD_VALUE))[3],0),

round(2000*(prop.table(table(ASA$FIELD_VALUE))[4]

+prop.table(table(ASA$FIELD_VALUE))[5]

+prop.table(table(ASA$FIELD_VALUE))[5]),0))

set.seed(2020)

#####

# ASA = 1

#####

ASAI <- ASA %>% filter(ASA$FIELD_VALUE=="I")

dim(ASAI)

ASA1_pats<- sample(1:dim(ASAI)[1], round(2000*prop.table(table(ASA$FIELD_VALUE))[1],0))

ASAI_Auswahl <- ASAI[ASA1_pats,]

dim(ASAI_Auswahl)

#####

# ASA = 2

#####

ASAII <- ASA %>% filter(ASA$FIELD_VALUE=="II")

dim(ASAII)

ASA2_pats<- sample(1:dim(ASAII)[1], round(2000*prop.table(table(ASA$FIELD_VALUE))[2],0)

)

ASAII_Auswahl <- ASAII[ASA2_pats,]

dim(ASAII_Auswahl)

#####

# ASA = 3

#####

ASAIII <- ASA %>% filter(ASA$FIELD_VALUE=="III")

dim(ASAIII)

ASA3_pats<- sample(1:dim(ASAIII)[1], round(2000*prop.table(table(ASA$FIELD_VALUE))[3],0) )

ASAIII_Auswahl <- ASAIII[ASA3_pats,]

dim(ASAIII_Auswahl)

#####

# ASA = 4

#####

ASAIV <- ASA %>% filter(ASA$FIELD_VALUE %in% c("IV"))

dim(ASAIV)

ASA4_pats<- sample(1:dim(ASAIV)[1], 20 )

ASAIV_Auswahl <- ASAIV[ASA4_pats,]

dim(ASAIV_Auswahl)

#####

# Auswahl OP IDs

#####

OP_ID_Auswahl <- c(ASAI_Auswahl$OP_ID,

ASAII_Auswahl$OP_ID,

ASAIII_Auswahl$OP_ID,

ASAIV_Auswahl$OP_ID)

length(OP_ID_Auswahl)

save(OP_ID_Auswahl,file="OP_ID_Auswahl.RData")
